# Production of d-Branched-Chain Amino Acids by Lactic Acid Bacteria Carrying Homologs to Isoleucine 2-Epimerase of *Lactobacillus buchneri*

**DOI:** 10.3389/fmicb.2018.01540

**Published:** 2018-07-13

**Authors:** Yuta Mutaguchi, Kano Kasuga, Ikuo Kojima

**Affiliations:** Department of Biotechnology, Faculty of Bioresource Sciences, Akita Prefectural University, Akita, Japan

**Keywords:** d-amino acid, branched-chain amino acid, epimerase, isoleucine 2-epimerase, lactic acid bacteria, racemase

## Abstract

Isoleucine 2-epimerase (ILEP) is a novel branched-chain amino acid racemase isolated from *Lactobacillus buchneri*. In this study, we examined production of free d-branched-chain amino acids such as d-valine, d-leucine, and d-*allo*-isoleucine, using lactic acid bacteria carrying homologs to ILEP. Twelve selected strains of lactic acid bacteria were grown at optimal growth temperatures and accumulation of d-branched-chain amino acids in the medium was monitored in exponential, early stationary, and stationary phases. To analyze the d-branched-chain amino acids, enantiomers in the medium were initially converted into diastereomers using pre-column derivatization with *o*-phthaldialdehyde plus *N*-isobutyryl-l-cysteine. The resultant fluorescent isoindole derivatives were analyzed on an octadecylsilyl stationary phase using ultra-high performance liquid chromatography. The analyses revealed that the seven following lactic acid bacteria carrying homologs showing 53–60% amino acid sequence identity to the *L. buchneri* ILEP accumulate d-branched-chain amino acids: *Lactobacillus fermentum* and *Weissella paramesenteroides* produce d-valine, d-leucine, and d-*allo*-isoleucine; *Lactobacillus reuteri, Leuconostoc mesenteroides* subsp. *mesenteroides*, and *Leuconostoc gelidum* subsp. *gasicomitatum* accumulate d-leucine and d-*allo*-isoleucine; and *Lactobacillus vaginalis* and *Leuconostoc pseudomesenteroides* produce d-*allo*-isoleucine. These results suggest that d-branched-chain amino acids are produced by a variety of lactic acid bacteria species, particularly those carrying homologs to the ILEP.

## Introduction

d-Amino acids, enantiomers of l-amino acids, play key roles as components of the peptidoglycan cell wall of bacteria. Within bacterial cell walls, d-alanine (d-Ala), and d-glutamate (d-Glu) are often the most common d-amino acids (Hancock, 1960; Schleifer and Kandler, 1972). In some lactic acid bacteria, which have a peptidoglycan type A4α or A4β, the peptidoglycans contain d-aspartate (d-Asp) in addition to d-Ala and d-Glu (Schleifer and Kandler, 1972; Bellais et al., 2006; Veiga et al., 2006). These d-amino acids are primarily produced from the corresponding l-enantiomers by enzymes such as alanine racemase (EC 5.1.1.1), glutamate racemase (EC 5.1.1.3), and aspartate racemase (EC 5.1.1.13). In our earlier study, marked accumulation of d-branched-chain amino acids (d-BCAAs) such as d-valine (d-Val), d-leucine (d-Leu), and d-*allo*-isoleucine (d-*allo*-Ile) was observed in the growth medium of the lactic acid bacterium *Lactobacillus otakiensis*. The enzyme activity responsible was detected as branched-chain amino acid racemase (BCAAR) activity including leucine racemase, valine racemase, and isoleucine 2-epimerase activities in *L. otakiensis* cells. On the basis of the *N*-terminal amino acid sequence of the purified enzyme, a gene encoding a homolog of this racemase from *L. otakiensis* was identified in the genome of *Lactobacillus buchneri* (Mutaguchi et al., 2013b). Although this gene is annotated as a γ-aminobutyrate aminotransferase (GABA-AT), the gene product enzyme expressed in *Escherichia coli* showed BCAAR activity, but not GABA-AT activity. The recombinant BCAAR, which is pyridoxal 5′-phosphate (PLP)-dependent, preferentially epimerizes between l-isoleucine (l-Ile) and d-*allo*-Ile, and also racemizes nonpolar amino acids including Val and Leu. This enzyme was therefore designated isoleucine 2-epimerase (ILEP) and assigned an identical EC number (EC 5.1.1.21). ILEP is a novel enzyme that catalyzes the racemization of BCAAs as its main substrate and seems to be involved in the production of d-BCAAs in lactic acid bacteria. In fact, homologous genes whose deduced amino acid sequence shows more than 30% identity with that of the *L. buchneri* ILEP are widely distributed among lactic acid bacteria. Elucidation the actual ability of d-BCAAs production by lactic acid bacteria carrying the ILEP homologous gene is extremely informative to reveal the physiological functions of d-BCAAs in lactic acid bacteria, thereby leading to the application of the amino acids production. In this study, therefore, we investigated the levels of d-BCAAs in growth media and the ILEP activity in cells of 12 strains of lactic acid bacteria carrying the ILEP homologous gene.

## Materials and methods

### Materials

*N*-Isobutyryl-l-cysteine (NIC) and *o*-phthaldialdehyde (OPA) were, respectively, purchased from Sigma-Aldrich Corp. LLC (St. Louis, MO, USA) and Nacalai Tesque Inc. (Kyoto, Japan). MRS medium was obtained from Becton, Dickinson and Co. (Franklin Lakes, NJ, USA). d-Amino acid oxidase from porcine kidney and catalase from bovine liver were purchased from Sigma.

### Microorganisms and growth conditions

Twelve strains of lactic acid bacteria used for this study and database information of their ILEP homologous gene are presented in Table 1. All strains were obtained from the Japan Collection of Microorganisms (JCM, Tsukuba, Japan) and the National Institute of Technology and Evaluation Biological Resource Center (NBRC, Tokyo, Japan). These strains were aerobically cultured in their recommended media at their respective optimum cultivation temperatures (Table 1). The recommended medium for *Oenococcus oeni* JCM 6125, 143 *Leuconostoc oenos* medium, was prepared according to the recipe published on the JCM website (http://www.jcm.riken.jp/cgi-bin/jcm/jcm_grmd?GRMD=143).

**Table 1:** Lactic acid bacterium strains used for analyzing the d-BCAAs production.

**Strain**	**ILEP homologous gene**			**Cultivation temperature**	**Culture medium**
	**Locus tag^a^**	**Annotation^a^**	**Identity^b^(%)**		
*Lactobacillus fermentum* NBRC 3956	LAF_1619	Aminotransferase	56	37°C	MRS
*Lactobacillus vaginalis* JCM 9505	HMPREF0549_0024	GABA-AT^c^	60	37°C	MRS
*Lactobacillus reuteri* JCM 1112	LAR_0189	GABA-AT	56	37°C	MRS
*Lactobacillus plantarum* subsp. *plantarum* JCM 1149	JCM1149DRAFT_00332	GABA-AT	71	30°C	MRS
*Lactobacillus ruminis* JCM 1152	Ga0074155_10338	GABA-AT	30	37°C	MRS
*Leuconostoc mesenteroides* subsp. *mesenteroides* JCM 6124	LEUM_0555	GABA-AT related aminotransferase	59	30°C	MRS
*Leuconostoc pseudomesenteroides* JCM 9696	EY9DRAFT_02771	GABA-AT	58	30°C	MRS
*Leuconostoc gelidum* subsp. *gasicomitatum* JCM 12535	LEGAS_1339	GABA-AT	56	22°C	MRS
*Streptococcus mutans* JCM 5705	Ga0069310_11641	AO-AT^d^	30	37°C	MRS
*Oenococcus oeni* JCM 6125	A3G9DRAFT_00945	GABA-AT	86	30°C	143 medium
*Enterococcus malodoratus* JCM 8730	UAI_00297	AO-AT	30	37°C	MRS
*Weissella paramesenteroides* JCM 9890	HMPREF0877_0835	GABA-AT	53	30°C	MRS

a Information on locus tags and annotation was cited from Integrated Microbial Genomes and Microbiomes (IMG/M).

b Identity to L. buchneri ILEP based on amino acid sequence.

c Abbreviation for γ-aminobutyrate aminotransferase.

d Abbreviation for acetylornithine aminotransferase.

### Preparation of culture media for d-amino acid analyses

For sequential analysis of the d-amino acid content of the culture medium conditioned by each of the lactic acid bacteria, sample solutions were prepared as follows. Each of strains was cultured to the stationary phase in 5 mL of the recommended medium. Aliquots (1 mL) of the culture medium were centrifuged (10,000 × *g* for 1 min at 4°C), and the supernatants were filtered through a centrifugal filter 3 K device (Amicon Ulta 0.5 mL, Merck Millipore, Darmstadt, Germany). The prepared samples were stored at −20°C until use.

### Analysis of l-amino and d-amino acids using UHPLC

d- and l-Amino acids in sample solutions were derivatized with OPA and NIC (Brückner et al., 1994) using an OPA-NIC solution prepared by dissolving 10 mg of OPA and 10 mg of NIC in 1 mL of methanol. The reaction mixture (250 μL) for the derivatization contained 5 μL of amino acid sample, 10 μL of OPA-NIC solution, and 35 μL of 0.4 M borate-sodium hydroxide buffer (pH 10.4). After derivatization for 2 min at 15°C in the dark, an aliquot (1 μL) of the reaction mixture was introduced into an ultra-high performance liquid chromatography (UHPLC) system (Nexera X2; Shimadzu Corp., Kyoto, Japan). The diastereoisomeric derivatives of amino acids formed with OPA-NIC were applied to a TSKgel ODS-12OH 2.0 mm I.D. × 15 cm column (Tosoh Corp., Tokyo, Japan) in the UHPLC. The UHPLC system consisted of a system controller CBM-20A, a solvent delivery unit LC-30AD, an HPLC column oven CTO-20AC, an autosampler SIL-30AC and a fluorescence detector RF-20Axs (all apparatus from Shimadzu Corp.). The excitation and emission wavelengths for fluorescent detection of the diastereoisomeric amino acid derivatives were, respectively, 230 and 445 nm. The data were processed using Labstations (Shimadzu). The system was operated at a flow rate of 0.25 mL/min at 25°C. The UHPLC gradient system for analysis of OPA-NIC derivatives (*A* = 50 mM sodium acetate, pH 5.9, *B* = methanol, and *C* = acetonitrile) was 12.2–22.8% B and 2.0–3.8% C for 6 min, 22.8% B and 3.8% C for 4 min, 22.8–33.4% B and 3.8–5.6% C for 2 min, 33.4% B and 5.6% C for 2 min, 33.4–35.5% B and 5.6–5.9% C for 0.25 min, 35.5% B and 5.9% C for 1.25 min, 35.5–41.4% B and 5.9–6.9% C for 0.5 min, 41.4% B and 6.9% C for 5 min, 41.4–49.3% B and 6.9–8.2% C for 3 min, 49.3% B and 8.2% C for 3.5 min, and 49.3–12.2% B and 8.2–2.0% C for 0.01 min. The peak area and retention time were, respectively, used for amino acid quantification and identification. Using OPA-NIC derivatization, 35 kinds of amino acids were analyzed simultaneously: d-form and l-form of Asp, Glu, Ala, Val, Leu, asparagine (Asn), glutamine (Gln), serine (Ser), methionine (Met), tryptophan (Trp), tyrosine (Tyr), phenylalanine (Phe), arginine (Arg), histidine (His), lysine (Lys), l-Ile, d-*allo*-Ile, l-threonine (l-Thr), d-*allo*-threonine (d-*allo*-Thr), and glycine (Gly). For this study, analysis was conducted with emphasis on d-BCAAs.

### d-amino acid oxidase treatment

Bacterial medium samples usually contain any unidentified amino compound that gives the same retention time-peak as a d-amino acid. Therefore, the peaks of d-amino acids, such as d-branched-chain amino acids, were confirmed by the reduction in fluorescence intensity elicited by treatment with d-amino acid oxidase (DAO) from *Sus scrofa* (Tosa et al., 1974). The DAO has very broad substrate specificity, oxidizing 12 d-amino acids including d-branched-chain amino acids (D'Aniello et al., 1993). Treatment with DAO removed the 12 susceptible d-amino acids from the medium samples, causing the respective peaks to be smaller than those obtained with the corresponding untreated samples. The DAO treatment was conducted as described in an earlier report (Mutaguchi et al., 2013a).

### Preparation of cell extract for BCAAR assay

For determination of BCAAR activity in lactic acid bacteria cells, cell extract was prepared as follows. Each of 12 strains was cultured until the end of exponential phase in 37.5 mL of recommended medium, and the cells were collected by centrifugation. The harvested cells were washed twice with 150 mM NaCl, suspended in 1 mL of 50 mM sodium-phosphate buffer (pH 7.2), disrupted using a multi-bead shocker (Yasui Kikai, Osaka, Japan), and centrifuged (10,000 × *g* for 30 min at 4°C). The resultant supernatant was used as the cell extract.

### Determination of BCAAR activity in the bacterial cells

BCAAR activity was assayed by measuring the amount of d-BCAAs resulted from l-BCAAs during the epimerization or racemization reaction. The reaction mixture (500 μL), consisting of 200 mM sodium-phosphate buffer (pH 7.0), 50 mM substrate (l-Val, l-Leu, or l-Ile), 0.1 mM PLP and 50 μL of the cell extract, was incubated at the same temperature as the optimum growth temperature of the strain. The reaction was stopped by addition of 125 μL of 50% trichloroacetic acid (TCA) to the reaction mixture when less than 20% of the substrate was consumed. The maximum reaction time was up to 24 h. After incubation for 5 min at 25°C, the mixture was centrifuged (10,000 × *g* for 15 min at 4°C). An aliquot (500 μL) of the supernatant was neutralized by addition of 300 μL of 1 M NaOH. The amount of product, d-BCAA, in the solution was measured using UHPLC as described above.

### Protein determination

Protein concentration was determined using the method described by Bradford (Bradford, 1976). Bovine serum albumin was used as the standard.

## Results

### Analysis of the d-BCAAs in the culture medium of 12 strains of lactic acid bacteria

**Figure 1:**
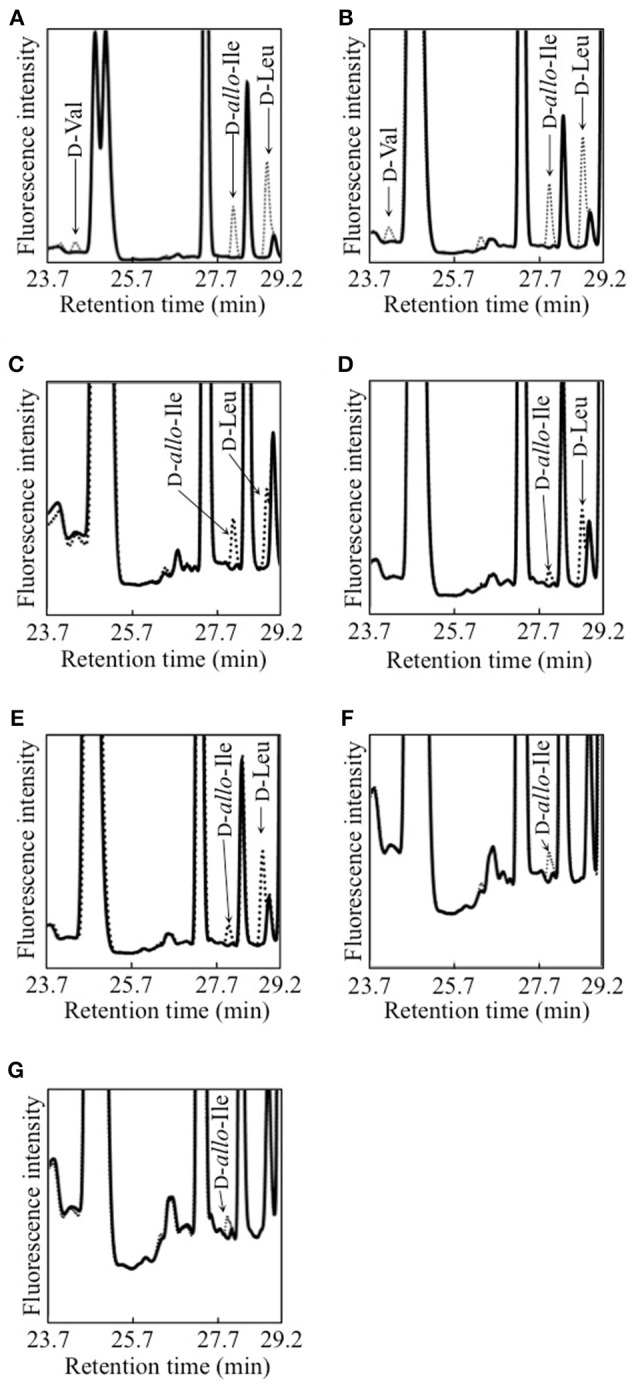
Detection of d-BCAAs in culture media of 7 strains of lactic acid bacteria. **(A)**, *L. fermentum*; **(B)** *W. paramesenteroides*; **(C)**, *L. reuteri*; **(D)**, *L. mesenteroides* subsp. *mesenteroides*; **(E)**, *L. gelidum* subsp. *gasicomitatum*; **(F)**, *L. vaginalis*; **(G)**, *L. pseudomesenteroides*. Dotted and solid lines show chromatograms of samples before and after DAO treatment, respectively.

Each of 12 strains of lactic acid bacteria carrying an *L. buchneri* ILEP homologous gene was cultured until the stationary phase, and accumulation of d-BCAAs in the culture medium was investigated. For d-Val, d-*allo*-Ile, and d-Leu, the UHPLC peaks were identified using retention times and confirmed to be derived from d-amino acids by DAO treatment. The results were the following: *Lactobacillus furmentum* and *Weissella paramesenteroides* produced d-Val, d-*allo*-Ile, and d-Leu (Figures 1A,B); *Lactobacillus reuteri, Leuconostoc mesenteroides* subsp. *mesenteroides*, and *Leuconostoc gelidum* subsp. *gasicomitatum* produced d-*allo*-Ile and d-Leu (Figures 1C–E); *Lactobacillus vaginalis* and *Leuconostoc pseudomesenteroides* produced only d-*allo*-Ile (Figures 1F,G). However, no d-BCAA was produced by *Lactobacillus ruminis, Streptococcus mutans, Enterococcus malodoratus, Oenococcus oeni*, nor *Lactobacillus plantarum* subsp. *plantarum*.

**Figure 2:**
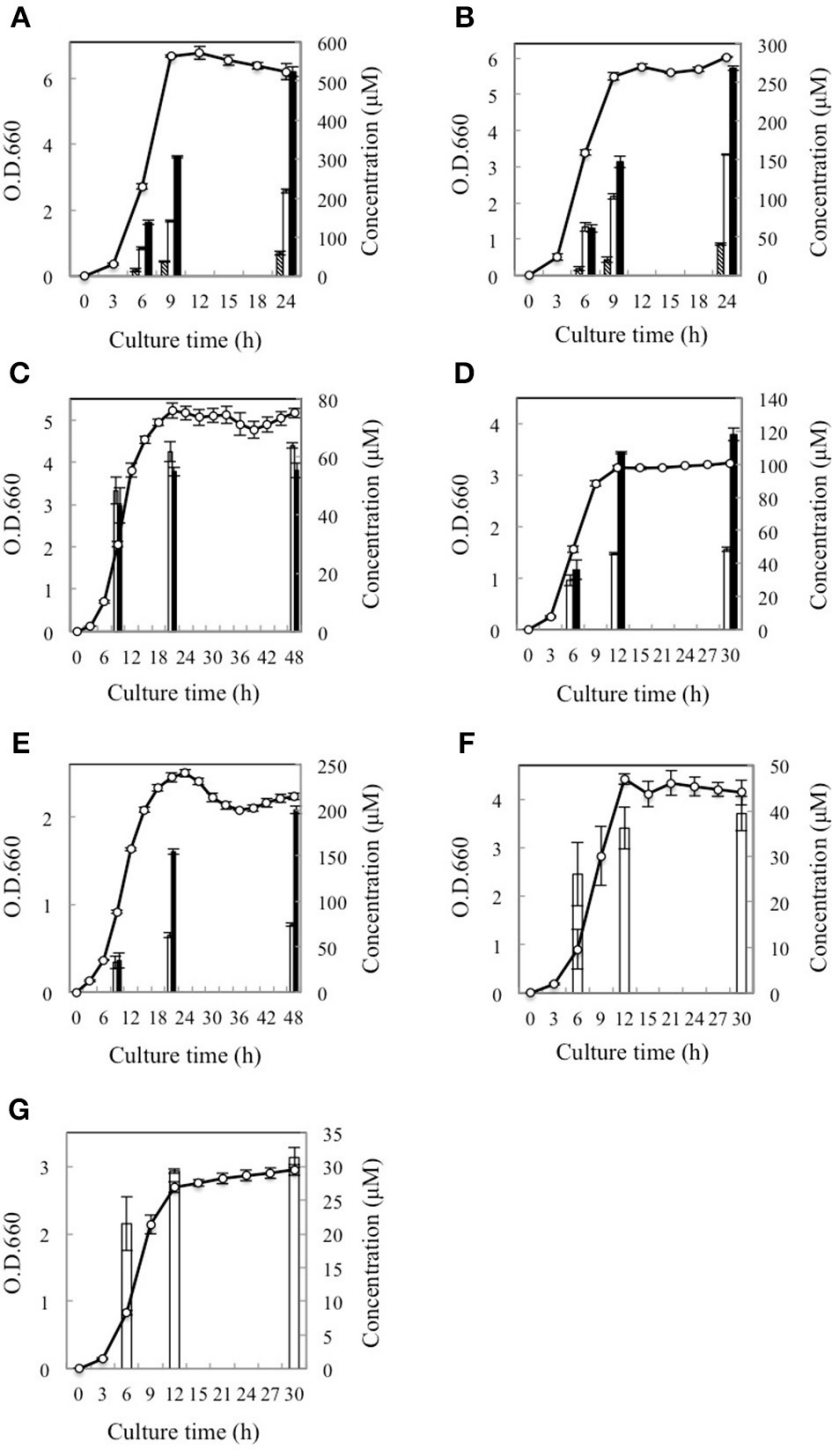
Time course of D-BCAA accumulation in culture media of 7 strains of lactic acid bacteria. **(A)**, *L. fermentum*; **(B)**, *W. paramesenteroides*; **(C)**, *L. reuteri*; **(D)**, *L. mesenteroides* subsp. *mesenteroides*; **(E)**, *L. gelidum* subsp. *gasicomitatum*; **(F)**, *L. vaginalis*; **(G)**, *L. pseudomesenteroides*. Solid bars, empty bars, and diagonal lines bars, respectively, show concentrations of d-Leu, d-*allo*-Ile, and d-Val. Cell concentrations (open circles) were assayed at O.D.600.

Subsequently, the d-BCAA concentrations in the culture media were measured in the middle and end of exponential phase and in the stationary phase for the d-BCAA producing seven strains. The results are portrayed in Figure 2 and d-BCAAs accumulated by the lactic acid bacteria are summarized in Table 2. The levels of d-Val, d-*allo*-Ile, and d-Leu produced by *L. fermentum* and *W. paramesenteroides* continued to increase until their stationary phase (Figures 2A,B). The levels of d-BCAAs produced by *L. reuteri, L. mesenteroides* subsp. *mesenteroides, L. gelidum* subsp. *gasicomitatum, L. vaginalis*, and *L. pseudomesenteroides*, however, increased until the end of exponential phase (Figures 2C–G). Results show that the remarkable increases in the d-BCAAs were occurred in the exponential phase and the d-BCAA levels continued to be almost constant through out the stationary phase.

**Table 2:** d-BCAA concentrations in the culture media in the stationary phase for the d-BCAA producing strains.

**Strain**	**Concentration (**μ**M)**^**a**^		
	**d-Val**	**d-*allo*-Ile**	**d-Leu**
*L. fermentum*	59.3 ± 5.0	219 ± 5	523 ± 13
*W. paramesenteroides*	40.1 ± 1.3	156 ± 6	268 ± 3
*L. reuteri*	−^b^	63.9 ± 0.9	55.4 ± 2.5
*L. mesenteroides* subsp. *mesenteroides*	−	48.6 ± 1.5	118 ± 4
*L. gelidum* subsp. *gasicomitatum*	−	74.1 ± 1.9	200 ± 4
*L. vaginalis*	−	39.4 ± 9.7	−
*L. pseudomesenteroides*	−	31.3 ± 1.5	−

a Values were obtained through repeated measurements (n = 3).

b –, not detected.

### Determination of BCAAR activity in the bacterial cells

In order to confirm that d-BCAAs accumulated in the culture media were derived from racemization or epimerization of BCAAs in the bacterial cells tested, BCAAR activity in the cells was determined using cell extracts of the 12 strains of lactic acid bacteria. Results show that BCAAR activity, which resulted in the accumulation of d-BCAA in culture medium, was detected in the cells of seven strains (Table 3). In addition, racemase activity for d-BCAAs not accumulated in the culture medium was also detected in the four strains: leucine racemase activity in *L. vaginalis* and *L. pseudomesenteroides*; valine racemase activity in *L. mesenteroides* subsp. *mesenteroides, L. gelidum* subsp. *gasicomitatum*, and *L. pseudomesenteroides*. However, no BCAAR activity was detected in the cell extracts of *L. ruminis, S. mutans, E. malodoratus, O. oeni*, nor *L. plantarum* subsp. *plantarum* after the incubation of reaction mixture for 24 h.

**Table 3:** BCAAR activity in the cells of d-BCAA producing lactic acid bacteria.

**Strain**	**Reaction time^a^ (h)**	**Specific activity (nmol/min/mg protein)**^**b**^		
		**l-Val^c^**	**l-Ile^c^**	**l-Leu^c^**
*L. fermentum*	1	1.97 ± 0.13	25.3 ± 2.07	23.4 ± 2.22
*W. paramesenteroides*	6	6.76 ± 0.91	33.5 ± 2.6	8.36 ± 0.84
*L. reuteri*	24	−^d^	0.198 ± 0.017	0.112 ± 0.013
*L. mesenteroides* subsp. *mesenteroides*	6	0.784 ± 0.040	2.46 ± 0.22	1.01 ± 0.09
*L. gelidum* subsp. *gasicomitatum*	2	2.83 ± 0.23	6.81 ± 0.52	0.756 ± 0.049
*L. vaginalis*	24	−	0.879 ± 0.065	1.48 ± 0.11
*L. pseudomesenteroides*	24	0.260 ± 0.017	1.07 ± 0.08	0.157 ± 0.014

a The actual reaction times for measurement of BCAAR activity.

b Values were obtained through repeated measurements (n = 3).

c Substrates used in triplicate reactions.

d –,not detected.

## Discussion

**Figure 3:**
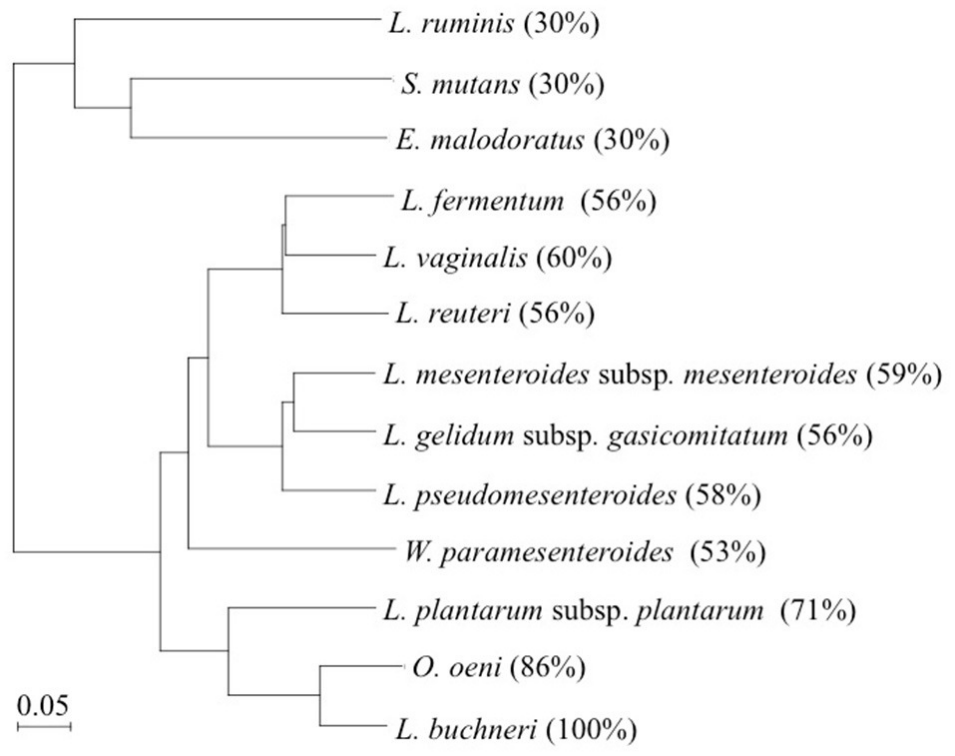
Phylogenetic tree of *L. buchneri* ILEP with ILEP homologs in 12 lactic acid bacteria. Numbers in parentheses denote identity with *L. buchneri* ILEP based on the amino acid sequence.

In this study, the production of d-BCAAs such as d-Val, d-*allo*-Ile, and d-Leu by 12 strains of lactic acid bacteria carrying a homologous gene to *L. buchneri* ILEP was investigated. Amino acid sequences of these homologous genes show more than 30% identity with that of the *L. buchneri* ILEP. These homologous genes are annotated as putative aminotransferases such as acetylornithine aminotransferase (AO-TA) and GABA-AT (Table 1). Specifically examining the phylogenetic tree based on the deduced amino acid sequences of the homologous genes, three clades were readily apparent (Figure 3). The first clade included homologs showing 30% identity with the *L. buchneri* ILEP. For strains carrying homologs included in the clade, neither accumulation of d-BCAAs in the growth medium nor BCAAR activity in the cell extract was detected. According to the genome database, the homologous gene of *L. ruminis* is annotated as a putative GABA-AT gene, with some γ-aminobutyrate metabolism-related genes lying around it. The homologous genes of *S. mutans* and *E. malodoratus* are annotated as a putative AO-AT gene, with some genes possibly related to acetylornithine metabolism around them. It is, therefore, considered that enzymes expressed from these homologous genes in *L. ruminis, S. mutans*, and *E. malodoratus* might be responsible for a function as an aminotransferase, but not as an ILEP.

Homologs showing between 53 and 60% identity with the *L. buchneri* ILEP were included in the second clade, and all the strains in this clade produced some of d-BCAAs and showed BCAAR activity in the cells. In addition, each of homologous genes in this clade neighbors a putative amino acid permease gene in each genome without any other neighboring genes. These facts suggest that enzymes expressed from these homologous genes belonging to the clade played a role in the same manner as *L. buchneri* ILEP, and the permease might be involved in d-BCAA production.

**Figure 4:**
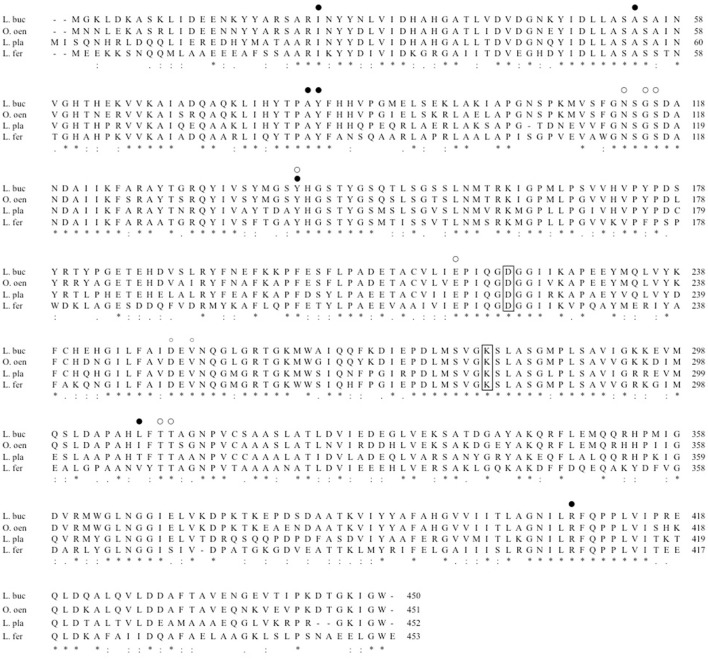
Multiple alignment of amino acid sequences of *L. buchneri* ILEP with ILEP homologs. L. buc, *L. buchneri*; O. oen, *O. oeni*; L. pla, *L. plantarum* subsp. *plantarum*; L. fer, *L. fermentum*. Lys residues abstracting α-hydrogen of l-form of the substrate and Asp residues adding α-hydrogen to the quinonoid intermediate are surrounded by lines. The substrate-binding and cofactor-binding residues are, respectively, marked by filled circles and open circles.

The third clade, to which *L. buchneri* ILEP belonged, consists of homologs conserved in genomes of *O. oeni* and *L. plantarum* subsp. *plantarum*. The homologs in the two strains showed more than 70% identity with the *L. buchneri* ILEP. In addition, the members in this clade also accompany a putative amino acid permease gene in the genome as observed in the second clade homologs. Despite the high identity, however, *O. oeni* and *L. plantarum* subsp. *plantarum* failed to produce d-BCAAs in the growth media and showed no BCAAR activity was detected in the cell extracts. Hayashi et al. reported that l-Ile epimerization by *L. buchneri* ILEP proceeds through abstraction of the α-hydrogen from the substrate by Lys280, whereas Asp222 serves as the catalytic residue adding an α-hydrogen to the quinonoid intermediate to form d-*allo*-Ile (Hayashi et al., 2017). Figure 4 shows the amino acid sequence alignment among ILEP homologs of the two bacteria, *L. fermentum*, and *L. buchneri* to reveal that the amino acid residues corresponding to Lys280 and Asp222 of *L. buchneri* ILEP were well conserved in the homologs of *O. oeni* (Lys280 and Asp222) and *L. plantarum* subsp. *plantarum* (Lys281 and Asp223), similarly in those of *L. fermentum*. In addition, most of substrate-binding residues and cofactor-binding residues of *L. buchneri* ILEP (Hayashi et al., 2017) were also conserved among the homologs. It is therefore crucial to clarify the reason for failure of d-BCAA production by the two lactic acid bacteria through analysis of the transcription levels of the ILEP homologous genes together with enzymatic characterization of the recombinant protein.

Lam et al. reported that *Vibrio cholerae* produced several d-amino acids including d-Leu and d-Val from the end of its logarithmic growth phase to the stationary phase. The bacterium used these d-amino acids for regulating cell wall remodeling during its stationary phase (Lam et al., 2009). In contrast, however, for the seven lactic acid bacteria used in this study, the production and accumulation of d-BCAAs started simultaneously with the start of the bacterial growth, and concentrations of d-BCAAs in the cultures increased along with the bacterial growth. To elucidate physiological functions of d-BCAAs in lactic acid bacteria, it is thus necessary to discuss the relation between d-BCAA production and growth of lactic acid bacteria.

Recently, many novel functions of bacterial d-amino acids have been reported except for those as peptidoglycan building blocks. For instance, Sasabe et al. reported that enterobacterial d-amino acids such as d-Ala, d-Asp, and d-Glu regulate gut microbiota of host mammalian via DAO from the host mammalian (Sasabe et al., 2016). Moreover, as described above, *Vibrio cholerae* are considered to use several d-amino acids for regulating cell wall remodeling during their stationary phase (Lam et al., 2009). These reports commonly show that free d-amino acids secreted from one bacterial cell directly or indirectly affect other bacterial cells. Free d-BCAAs produced by lactic acid bacteria might also have effects on other bacterial cells of the same or different species present in the surrounding environment. Among the lactic acid bacteria producing d-BCAAs examined for this study, *L. fermentum, L. vaginalis*, and *L. reuteri* are human indigenous bacteria isolated from mouth, vagina, and intestine, respectively (Lerche and Reuter, 1962; Embley et al., 1989; Dellaglio et al., 2004). The results of this study, therefore, indicate that d-BCAAs from these lactic acid bacteria might be related to human health through their influences on microbiota in human tissues. Furthermore, many kinds of d-amino acids including d-BCAAs are known to be much sweeter than the corresponding l-amino acids (Solms et al., 1965; Schiffman et al., 1981; Kawai et al., 2012). This study showed that lactic acid bacteria isolated from foods such as *L. mesenteroides* subsp. *mesenteroides, L. pseudomesenteroides, L. gelidum* subsp. *gasicomitatum*, and *W. paramesenteroides* (Collins et al., 1993; Björkroth et al., 2000) produced d-BCAAs, which might affect the taste of foods.

Although little has been clarified about free d-BCAAs production and functions in all organisms, this study shows d-BCAAs production by several lactic acid bacteria that are closely related to human life and indicates the importance of further studies focusing on the function of the amino acids in human health and food quality.

## Author contributions

YM planned this research and did all the practical experiment of this research. KK and IK contributed conception of this study and advised about organization of this manuscript.

### Conflict of interest statement

The authors declare that the research was conducted in the absence of any commercial or financial relationships that could be construed as a potential conflict of interest.
